# Probiotic Yeast and How to Use Them—Combining Traditions and New Waves in Fermented Beverages

**DOI:** 10.3390/foods14162921

**Published:** 2025-08-21

**Authors:** Adam Staniszewski, Patrycja Staniszewska, Elwira Komoń-Janczara, Monika Kordowska-Wiater

**Affiliations:** 1 Department of Biotechnology, Microbiology and Human Nutrition, University of Life Sciences in Lublin, Skromna 8, 20-704 Lublin, Poland; adam.staniszewski@up.lublin.pl (A.S.); elwira.komon.janczara@up.lublin.pl (E.K.-J.); 2 Department of Invertebrate Ecophysiology and Experimental Biology, University of Life Sciences in Lublin, Doświadczalna 50a, 20-280 Lublin, Poland; patrycja.staniszewska@up.lublin.pl

**Keywords:** probiotic yeast, podpiwek, underbeer, *Saccharomyces boulardii*, *Hanseniaspora uvarum*, *Metschnikowia pulcherrima*, *Saccharomyces cerevisiae* var boulardii, *Saccharomyces cerevisiae*, *Pichia kudriavzevii*, fermented beverages, functional food

## Abstract

Potentially probiotic yeasts isolated from foodstuffs can be used as components in functional fermented beverages. To date, there have been no reports on the use of *Saccharomyces cerevisiae* var. *boulardii*, *Pichia kudriavzevii*, *Metschnikowia pulcherrima*, or *Hanseniaspora uvarum* isolates in the production of a traditional Polish beverage called underbeer (podpiwek). The aim of the study was to determine the usefulness of six isolates of the above-mentioned species as starter cultures for the fermentation of underbeer. First, the important characteristics of the yeasts, like ethanol tolerance and H_2_S production, were examined. In the next stage, the wort was fermented by the tested yeasts, and cell viability, fermentation vigor, sugar assimilation, and production of metabolites, as well as properties of the beverage (pH, titratable acidity, color, and turbidity), were determined. Saccharomyces yeasts tolerated the addition of ethanol up to 16% (*v*/*v*), while *Pichia*, *Metschnikowia*, and *Hanseniaspora* tolerated up to 10% (*v*/*v*) ethanol, and all except *H. uvarum* produced H_2_S. The yeasts remained viable in the beverages for 1 month at the required level, utilized glucose, fructose and partially complex carbohydrates, and produced ethanol (*S. cerevisiae*, *P. kudriavzevii*, and *M. pulcherrima*) and organic acids such as tartaric, malic, and citric acid. The underbeers became sour and showed varying turbidity and a color corresponding to pale-amber beers. All tested strains produced fermented beverages that were low- or non-alcoholic with different properties. This experiment may be a starting point for research into regional products as probiotic or synbiotic foods; however, further research is required for selection of the best strains for underbeer fermentation.

## 1. Introduction

Fermented beverages have accompanied humanity since the dawn of time and have remained an important part of the human diet all over the world, and within such, the most commonly used beverages are wine, cider, kombucha, and beer [1,2,3,4,5].

The key stage in the production of this type of beverage is fermentation, often carried out by yeast, bacteria, or their co-cultures. The genera of yeasts most commonly found in fermented foods and beverages include *Debaryomyces*, followed by *Candida*, *Saccharomyces*, *Pichia*, *Kluyveromyces*, *Wickerhamomyces*, *Torulaspora*, *Yarrowia*, and *Metschnikowia* [6,7,8]. Several examples of the use of the commercial probiotic yeast *S. cerevisiae* var. *boulardii*, either alone or in co-cultures, for the production of probiotic beer based on cereal wort containing hops and other additives can be found in the scientific literature [9,10,11,12,13]. However, apart from the commonly used commercial species and strains (i.e., *Saccharomyces cerevisiae*), recent research has shown the potential of various yeast species, including probiotic strains belonging to the genera *Kluyveromyces*, *Debaryomyces*, *Candida*, *Pichia*, *Hanseniaspora*, and *Metschnikowia*, isolated from numerous sources and suggests the possibility of the usage of these non-conventional yeasts as inoculum in the fermentation of novel fermented food and beverages [6,7,14,15]. There are also reports of the use of unconventional yeasts in co-cultures with other yeasts, including Saccharomyces or lactic acid bacteria, to produce innovative fermented beverages such as craft beers, bitter beers, ciders, and others [16,17,18,19,20,21]. Numerous species of the aforementioned yeasts have been classified as moderate or weak fermenters, so they can potentially be used to produce non-alcoholic or low-alcohol beverages [22].

*P. kudriavzevii* was found in the fermentation of some alcoholic beverages, including beer, fermented fruits or berries, and traditional cereal beverages [23].

*Metschnikowia* spp. and *Hanseniaspora* spp. play a significant role in winemaking during spontaneous fermentation, as well as in fruit wine fermentation [24,25].

Hence, we arrived at the idea of combining two aspects important in the human diet: the consumption of beverages with desirable taste values and the addition of live cultures of organisms with probiotic potential. This combination makes such fermented beverages into functional foods according to the definition proposed by Temple, 2022, stating that “Functional foods are novel foods that have been formulated so that they contain substances or live microorganisms that have a possible health-enhancing or disease-preventing value, and at a concentration that is both safe and sufficiently high to achieve the intended benefit. The added ingredients may include nutrients, dietary fiber, phytochemicals, other substances, or probiotics” [26]. Nowadays, the development of plant-based functional foods including cereals is a topic of interest due to their content of vitamins, minerals, and dietary fiber [12].

As a continuation of our previous research on the isolation of yeasts with probiotic potential, we decided to combine tradition with modernity and attempted to produce the Polish traditional fermented beverage “podpiwek” using strains with probiotic potential that we had previously isolated [14,15]. The English name of the “podpiwek” beverage is not well established and is present in various sources in untranslated and translated forms [27,28]; hence, for the purposes of the article, we decided to use the term “underbeer”, which is the literal translation from the Polish language.

Underbeer is considered traditional to Poland and Lithuania and may date back to the times of the Polish-Lithuanian Commonwealth. Its first commercial production began in the mid-19th century. This drink was very popular until the end of the 1980s as an alternative to commercially available sweetened drinks, which were largely not easily accessible during the times of socialism. Underbeer is a fermented beverage made from roasted barley grains, known as cereal coffee, and ground chicory root with the addition of hops. The beverage does not contain malt. During fermentation, similar to the production of beer, yeasts assimilate sugar and produce alcohol and carbon dioxide, which means that the drink does not need to be artificially enriched with carbon dioxide. The finished drink has a slightly sour, refreshing taste with a hint of bitterness and a distinctive aroma of cereal coffee and yeast. Furthermore, due to its intense flavor, color and aroma, obtained through traditional production methods, this beverage does not require the addition of chemical preservatives, flavorings or colorings [3,27,28,29].

The aim of the research is to evaluate the usefulness of selected probiotic yeast strains as potential starter cultures for underbeer fermentation in terms of cell survival, sugar utilization, and production of metabolites influencing the physicochemical properties of beverages. Additionally, important characteristics of the strains used as starter cultures, such as ethanol tolerance and H_2_S production, were examined.

## 2. Materials and Methods

### 2.1. Strains and Inoculum Preparation

As starter cultures, the six yeast isolates with probiotic potential, originating from various types of fermented food and beverages, were obtained during our previous work [14,15]. As the positive control, we used *Saccharomyces cerevisiae* var. *boulardii* CNCM I-745 (Enterol, Biocodex, Gentilly, France).

The cultures were stored frozen in 30% glycerol at −80 °C, and before usage, the strains were cultured for 72 h at 28 °C in YPD broth (BTL, Łódź, Poland).

More detailed data about each strain (code names for individual strains, species) are included in Table 1.

To ensure uniformity of measurement, the original optical density at 600 nm (OD_600_) was determined for all cultured strains. Before the inoculation of the underbeers, liquid yeast cultures in YPD medium, after incubation at 28 °C for 48 h, were centrifuged for 10 min at 8000 rpm, washed, and the cell biomass was suspended in sterile water. The suspensions were then normalized to an OD_600_ of 1.0, and 2% (*v*/*v*) of such was used as inoculum for underbeer preparation.

### 2.2. Underbeer Preparation

In order to ensure homogeneity and limit the influence of raw materials on the properties of the tested underbeer, a decision was made to use a commercial underbeer base, “Podpiwek” (Bakalland S.A., Warsaw, Poland); the base includes the following: roasted sugar beet, barley, and chicory, hops, and acidity regulators (diphosphates and citric acid; 0.15% (±0.05%)). The wort was prepared according to the manufacturer’s recommendations with the addition of 500 g of sucrose for every 10 l of wort; wort was poured into 500 mL conical flasks, sterilized, inoculated in sterile conditions, and rubber stoppers with fermentation tubes were fitted on. Fermentations were carried out over 14 days at room temperature, and then the flasks were moved to fridge. The samples were taken on days 0 (control), 7, 14, and 30.

### 2.3. Properties of Strains Related to Beverage Production

#### 2.3.1. Ethanol Tolerance

The strains were cultured at 28 °C for 48 h in YPD broth with addition of different concentrations of ethanol. The ethanol concentration for each species was selected based on the scientific literature [30,31,32,33,34].

For *Saccharomyces cerevisiae* and *Saccharomyces cerevisiae* var. *bouardi* (strains 37_Sacch_cerevisiae and Sacch_boulardi), the ethanol concentration used were 8, 10, 12, 14, and 16% of EtOH (*v*/*v*). For the rest of the species, the ethanol concentrations were 2, 4, 6, 8, and 10% of EtOH (*v*/*v*). Control cultures of each strain were grown in YPD medium without ethanol. The growth of the tested strains was evaluated spectrophotometrically at OD_600_. The survivability (S) of yeast was calculated according to the following formula:

$$S=\frac{{OD}_{600}\;sample}{{OD}_{600}\;control}\times 100\%$$

#### 2.3.2. H_2_S Production

The tested strains were inoculated with a loop on Biggy Agar (Biomaxima, Lublin, Poland) in Petri dishes and incubated at 28 °C for 48 h. The growth of colonies and their change in the color to brown or black indicates the ability to produce H_2_S [35,36].

### 2.4. Parameters Related to Fermentation

#### 2.4.1. Yeast Viability in Fermented Beverages

In order to assess the number of live yeast cells in underbeer, serial dilutions of the samples were prepared in saline solution and 0.2 mL of each dilution was spread on Petri dishes with YGC (yeast extract, glucose, chloramphenicol) agar (BTL, Łódź, Poland) on days 0, 7, 14, and 30 and incubated at 28 °C for 72 h. After incubation, the colonies were counted and their numbers were converted to 1 mL of beverage [37].

#### 2.4.2. pH

The pH values were determined using a pH meter HI2210-02 (Hanna Instruments, Olsztyn, Poland). The pH of the wort at the beginning was 6.71.

#### 2.4.3. Titratable Acidity

Total titratable acidity (TTA) was determined according to Neffe-Skocińska et al., 2017 and Oliveira Alves et al., 2025 [38,39]. The samples were prepared by 10-fold dilution of underbeer (10 mL of underbeer, 90 mL of distilled water) and titrating 100 mL of the samples with 0.01 M NaOH. The value was expressed in g of acetic acid equivalent per liter (g/L) and was calculated from the following equation:

TTA = ((V_NaOH_ × 0.01 × 60.05)/V) × 10

where

V_NaOH_ = volume of NaOH used in the titration (in mL);

0.01 = molarity of the NaOH solution used in titration (in mol/L);

60.05 = equivalent weight of acetic acid (60.05 g/mol);

V = volume of sample titrated (in mL);

10 = dilution factor.

#### 2.4.4. Fermentative Vigor

The fermentative vigor (FV) was evaluated as the weight loss in g of CO_2_ to the atmosphere during fermentation. The fermentation vessels were weighed at the beginning of fermentation and days seven and fourteen [36].

#### 2.4.5. ASBC Turbidity

The American Society of Brewing Chemists Turbidity (ASBC Turbidity) was evaluated at time 0, 7, and 14 days according to the methodology used by Pyrovolou et al., 2024 [40].

#### 2.4.6. ASBC Color

Color measurement was performed at time 0, 7, and 14 days according to the methodology used by Pyrovolou et al., 2024 [40]. The EBC (European Brewery Convention) scale is used to express color units. The color value in EBC units is determined using the following formula:

Beer Color (EBC) = 25 × A_430_.

#### 2.4.7. High-Performance Liquid Chromatography Analysis

Samples collected after 7, 14, and 30 days were filtered using 0.22 µm pore size PTFE membrane syringe filters and subsequently diluted with distilled water. The quantification of post-fermentation products and substrate residues was conducted on the HPLC system Dionex UltiMate 3000 (Thermo Scientific, Mundelein, IL, USA) comprised of a pump (LPG-3400SD), an autosampler (WPS-3000SL), a column oven (TCC-3000SD), and Aminex HPX-87H column (300 × 7.8 mm, Bio-Rad, Hercules, CA, USA). A measure of 3 mM sulfuric acid aqueous solution was used as the mobile phase. A 20 μL sample was analyzed on a column maintained at 60 °C with a flow rate of 0.5 mL/min. The absorbance of organic acids at 210 nm was monitored using a UV-Vis detector (Dionex Ultimate, Thermo Scientific), while a refractometric detector (RefractoMax 521, Thermo Scientific, Mundelein, IL, USA) was employed to identify carbohydrates, ethanol, and glycerol [41]. Chromatograms were analyzed using the Chromeleon 7.3 software suite (Thermo Scientific, Mundelein, IL, USA).

### 2.5. Statistical Analysis

Statistical analysis of the obtained results was carried out using Statistica ver. 13.3 (2017) for Windows (StatSoft Inc., Tusla, OK, USA). Two-way ANOVA followed by Tukey HSD post hoc tests (*p* < 0.05) were used to compare the results for ethanol tolerance (2–10% EtOH in 24 and 48 h) for strains no. 15, 16, 101, 110, and 113, yeast viability (for 0, 7, 14, and 30 days), underbeer pH (7, 14, and 30 days), total titratable acidity (TTA) (for 7, 14, and 30 days), fermentative vigor (7 and 14 days), turbidity (0, 7, and 14 days) and color (0, 7, and 14 days) for each strain. One-way ANOVA was used to evaluate the level of ethanol tolerance (8–16% EtOH in 24 and 48 h) for strains no. 37 and SB and concentrations of sugars, ethanol, and organic acids for each strain (for 0, 7, 14, and 30 days). Detailed results (statistical significance) of the analyses are included in the Supplementary Materials.

## 3. Results

### 3.1. Properties of Strains Related to Beverage Production

#### 3.1.1. Ethanol Tolerance

**Figure 1:**
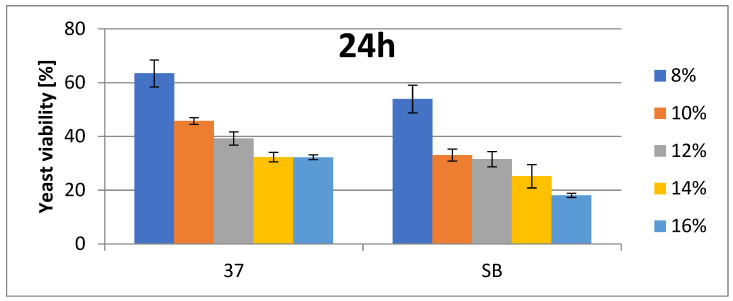
Ethanol tolerance for Saccharomyces spp. strains (37 and SB) at various EtOH concentrations after 24 h. Survivability of tested strains no. 37 and SB within 24 h in 8, 10, 12, 14, and 16% EtOH (two-way ANOVA: strain: F(1,20) = 72,703, *p* = 0.00000, se ± 0.004721; EtOH %: F(4,20) = 20,349, *p* = 0.00000; se ± 0.007465; strain × EtOH %: F(4,20) = 25,313; *p* = 0.00000, se ± 0.0.010557). Statistical significance of the analyses is included in the Supplementary Spreadsheet S1.

**Figure 2:**
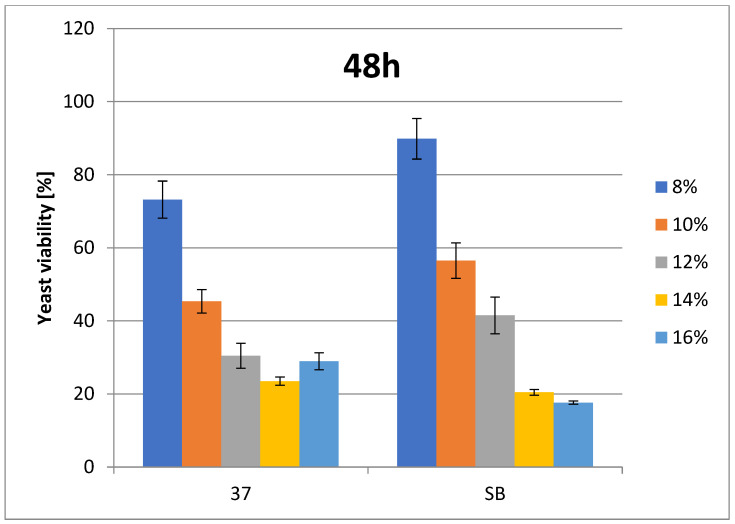
Ethanol tolerance for Saccharomyces spp. strains (37 and SB) at various EtOH concentrations after 48 h. Survivability of tested strains no. 37 and SB within 48 h in 8, 10, 12, 14, and 16% EtOH (two-way ANOVA: strain: F(1,20) = 83,267, *p* = 0.00000, se ± 0.005503; EtOH %: F(4,20) = 24,112, *p* = 0.00000; se ± 0.008701; strain × EtOH %: F(4,20) = 41,1991; *p* = 0.00000, se ± 0.012305). Statistical significance of the analyses is included in the Supplementary Spreadsheet S1.

**Figure 3:**
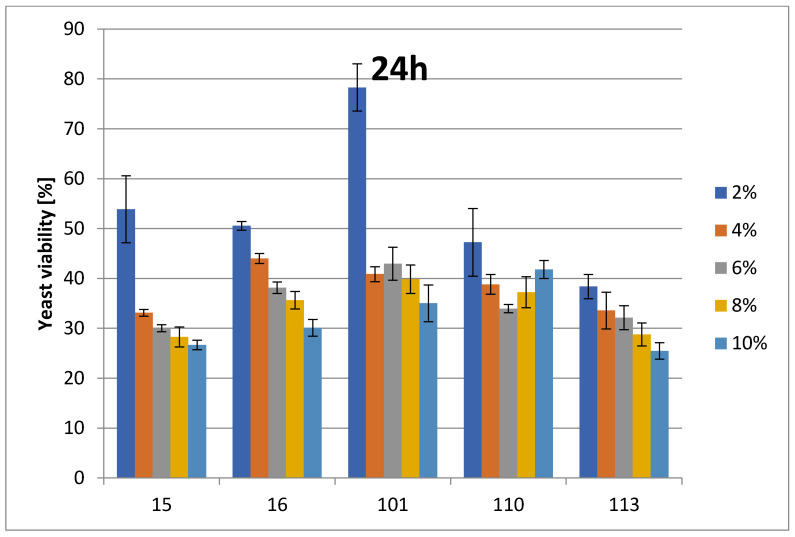
Ethanol tolerance for non-Saccharomyces strains at various EtOH concentrations after 24 h. Survivability of tested strains no. (15, 16, 101, 110, and 113) within 24 h in 0, 2, 4, 6, 8, 10, and 12% EtOH (two-way ANOVA: EtOH %: F(5,62) = 27,461, *p* = 0.00000; se ± 0.006318–0.014128; strain × EtOH %: F(20,62) = 25,563; *p* = 0.00000, se ± 0.014128; One-Way ANOVA: strain: F(4,88) = 5.2884, *p* = 0.00073, se ± 0.063141–0.068200). Statistical significance is included in the Supplementary Spreadsheet S1.

**Figure 4:**
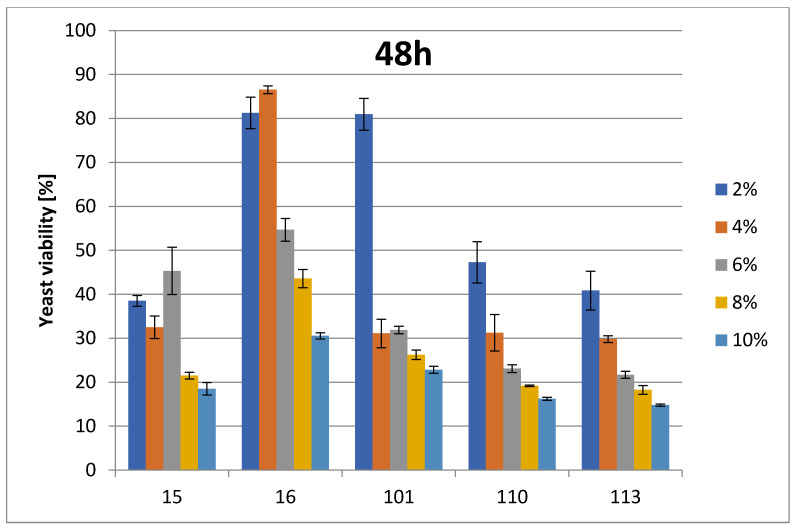
Ethanol tolerance for non-Saccharomyces strains at various EtOH concentrations after 48 h. Survivability of tested strains no. (15, 16, 101, 110, and 113) within 48 h in 0, 2, 4, 6, 8, 10, and 12% EtOH (two-way ANOVA:; EtOH %: F(5,62) = 117.35, *p* = 0.00000, se ± 0.006299–0.014084; strain × EtOH %: F(20,62) = 40,080, *p* = 0.00000, se ± 0.014084; One-Way ANOVA: strain: F(4,88) = 4.5875, *p* = 0.00206, se ± 0.079084–0.085420). Statistical significance of the analyses is included in the Supplementary Spreadsheet S1.

The change in cell number is presented as a percentage of growth decrease relative to the control at the appropriate ethanol concentrations. For *Saccharomyces* spp. (strains 37_Sacch_cerevisiae and Sacch_boulardi), ethanol concentrations were in the range of 8–16% (*v*/*v*). For the rest of the species, ethanol concentrations were in the range of 2–12% (*v*/*v*). The ethanol tolerances of Saccharomyces yeasts are presented at Figure 1 and Figure 2. The strain with the highest ethanol tolerance is the strain 37_Sacch_cerevisiae, still presenting about 32 and 23–29% survivability at 14 and 16% ethanol concentration after 24 and 48 h, respectively; the next-highest is the strain Sacch_boulardi, which presents 25 and 18% for 14 and 16% of ethanol after 24 h, with a slight decrease following after 48 h. In the case of a non-Saccharomyces yeast, 101_Pich_kudriavzevii showed the highest survival rate after 24 h of cultivation in the presence of 2, 6, and 8% ethanol, while after 48 h, 16_Hans_uvarum showed higher survival rates in the presence of all tested concentrations of added ethanol. Overall, even in the presence of 10% ethanol, yeasts survived at a rate of 25.5–35% after 24 h and 14.8–22.8% after 48 h, as is presented in Figure 3 and Figure 4.

#### 3.1.2. H_2_S Production

All tested strains with exception of 15_Hans_uvarum and 16_Hans_uvarum presented the ability to produce H_2_S (brown and dark brown colonies on Biggy Agar).

### 3.2. Parameters Related to Fermentation

#### 3.2.1. Yeast Viability in Fermented Beverages

**Figure 5:**
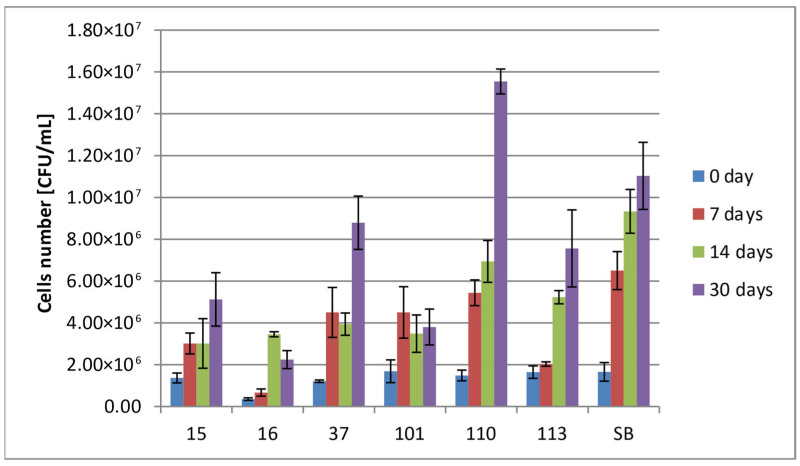
Yeast viability at day 0, 7, 14, and 30. Survivability of tested strains no. (15, 16, 37, 101, 110, 113, and SB) within 0, 7, 14, and 30 days (two-way ANOVA: strain: F(5,60) = 15,718, *p* = 0.0000, se ± 494,907.0; time: F(3,60) = 49,091, *p* = 0.0000; se ± 386,886.6; strain × time: F(15,60) = 4.1785; *p* = 0.00003, se ± 699,904.1–989,813.9). Statistical significance of the analyses is included in the Supplementary Spreadsheet S1.

The viability of the yeast in the wort was assessed at days 0, 7, 14, and 30. Generally, all of the strains presented growth within tested fermentation time, although some strains (16_Hans_uvarum, 37_Sacch_cerevisiae, 101_Pich_kudriavzevii) presented a temporary, slight decrease in the number of cells. The results presented in Figure 5 indicate that all strains retained their viability above 10^6^ CFU/mL throughout the entire analysis period.

#### 3.2.2. pH

**Figure 6:**
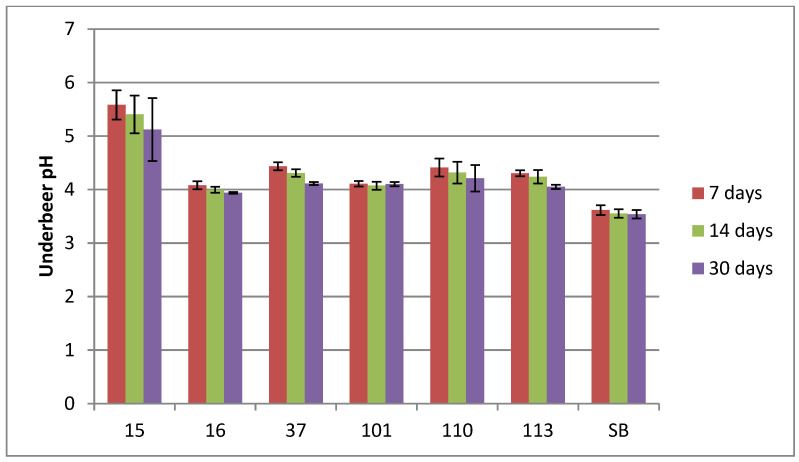
Underbeer at day 0, 7, 14, and 30. pH of tested strains no. (15, 16, 37, 101, 110, 113, and SB) within 7 and 14 day (two-way ANOVA: strain: F(5,45) = 49,379, *p* = 0.0000, se ± 0.082286; time: F(2,45) = 4.5594, *p* = 0.01574; se ± 0.055708; strain × time: F(10,45) = 0.37153; *p* = 0.95266, se ± 0.142523). Statistical significance of the analyses is included in the Supplementary Spreadsheet S1.

The pH results for whole period of fermentation are presented at Figure 6. The pH decreased during the fermentation period for all tested strains, with the highest pH value in 15_Hans_uvarum and the lowest pH value in Sacch_boulardi.

#### 3.2.3. Titratable Acidity

All the strains showed an increase in total titratable acidity (TTA) within the duration of the experiment. The lowest TTA value was determined for the beverage made with the strain 15_Hans_uvarum and the highest TTA value was in the beverage produced by Sacch_boulardi during the experiment. The TTA ranged from 0.094 g of acetic acid equivalent per liter for strain 15_Hans_uvarum to 0.97 g/L for control Sacch_boulardi at day 7. For day 14 TTA ranged from 0.102 g/L of acetic acid equivalent per liter for strain 15_Hans_uvarum up to 1.02085 g/L for the control Sacch_boulardi. At day 30, the TTA ranged between 0.114 g/L for the strain 15_Hans_uvarum and 1.085 g/L for the SB.

**Figure 7:**
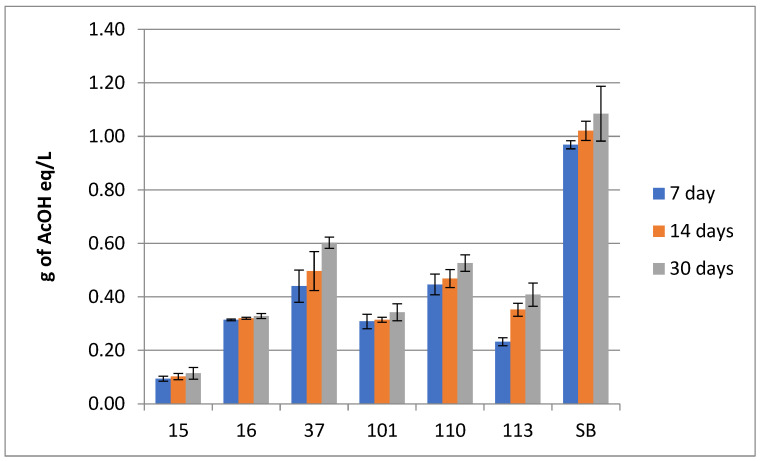
Total titratable acidity (TTA) of beverages produced by the tested strains. TTA of tested strains no. (15, 16, 37, 101, 110, 113, and SB) within 7, 14, and 30 days (two-way ANOVA: strain: F(5,24) = 5.0736, *p* = 0.00259, se ± 0.070830–0.100168; time: F(2,24) = 0.20727, *p* = 0.81424; se ± 0.067814; strain × time: F(10,24) = 0.06997; *p* = 0.99994, se ± 0.173497). Statistical significance of the analyses is included in the Supplementary Spreadsheet S1.

The results of the assay are presented in Figure 7.

#### 3.2.4. Fermentative Vigor

The fermentative vigor (FV) was evaluated as weight loss expressed as g of CO_2_ (*v*/*v*) per 100 mL of wort. The fermentation vessels were weighted at the beginning of fermentation and days 7 and 14. The difference in fermentative vigor was strain-dependent and increased during the fermentation period. The strain 15_Hans_uvarum presented the lowest FV both at 7 d and 14 d (acc. 0.357 g and 0.557 g). Conversely, the control strain, Sacch_boulardi, presented the highest FV on days 7 and 14 (acc. 6.587 g and 10.83 g).

**Figure 8:**
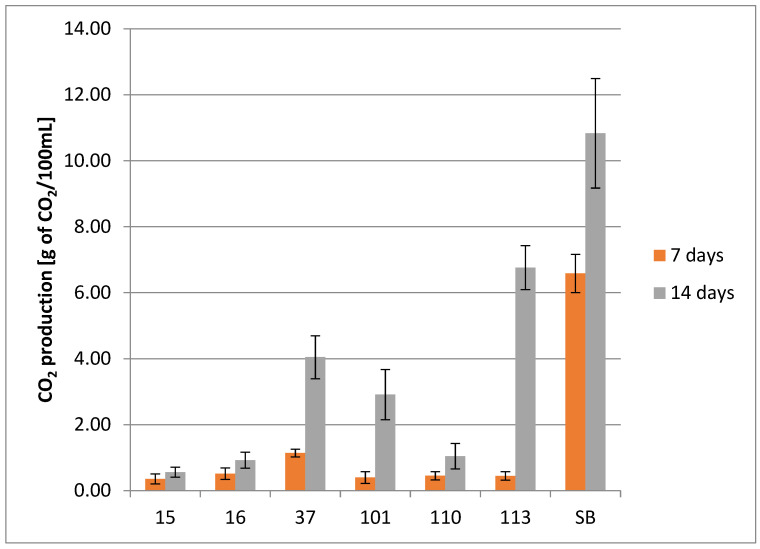
Fermentative vigor of the tested yeast strains. Fermentative vigor of tested strains no. (15, 16, 37, 101, 110, 113, and SB) within 7 and 14 days (two-way ANOVA: strain: F(6,28) = 46,242, *p* = 0.00000, se ± 0.427739; time: F(1,28) = 57,659, *p* = 0.00000; se ± 0.228636; strain × time: F(6,28) = 7.0805; *p* = 0.00012, se ± 0.604914). Statistical significance of the analyses is included in the Supplementary Spreadsheet S1.

The results of the assay are presented in Figure 8.

#### 3.2.5. ASBC Turbidity

**Figure 9:**
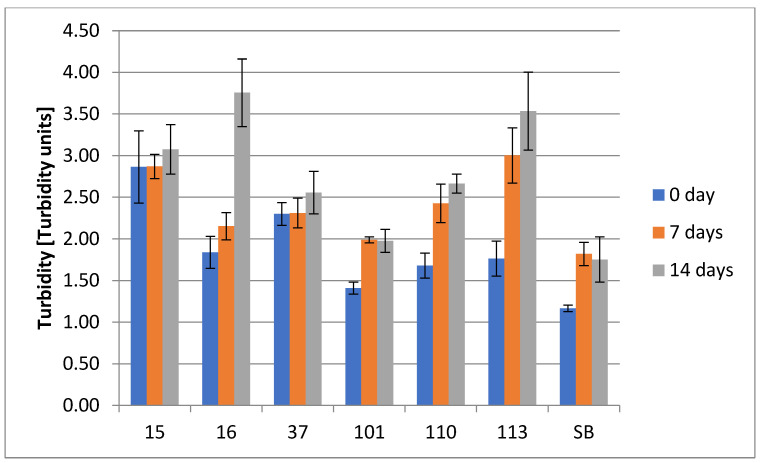
ASBC Turbidity of the tested underbeers. Results of the turbidity of tested strains no. (15, 16, 37, 101, 110, 113, and SB) within 0, 7, and 14 days (two-way ANOVA: strain: F(5,45) = 44,535, *p* = 0.0000, se ± 0.081080; time: F(2,45) = 17,763, *p* = 0.0000, se ± 0.054891; strain × time: F(10,45) = 21,912; *p* = 0.0000, se ± 0.099302–0.140435). Statistical significance of the analyses is included in the Supplementary Spreadsheet S1.

The results of turbidity assay at days 0, 7, and 14 are presented in Figure 9. Almost all the tested strains presented increases in turbidity within the whole fermentation period, except the strains 15_Hans_uvarum, 37_Sacch_cerevisiae, and Sacch_boulardi. In the case of the strains 15_Hans_uvarum and 37_Sacch_cerevisiae, turbidity remained quite similar for the entire fermentation period, although 101_Pich_kudriavzevii and Sacch_boulardi showed slight decreases in turbidity at day 14.

#### 3.2.6. ASBC Color

**Figure 10:**
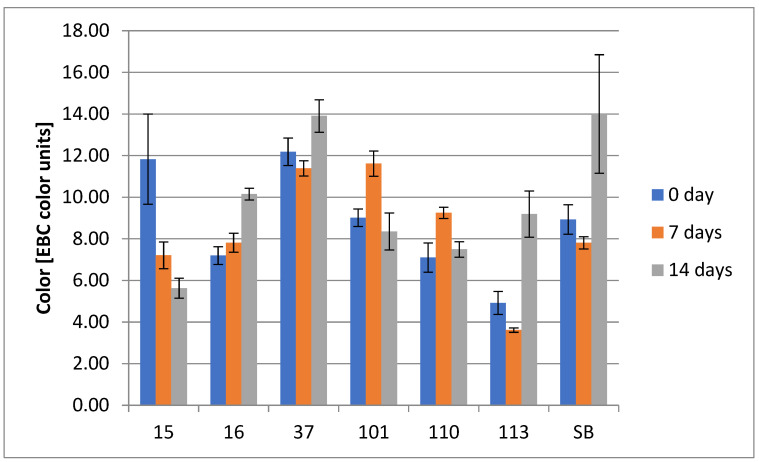
ASBC Color (EBC units) of the underbeers produced by the selected yeast strains. Results in the color assay of tested strains no. (15, 16, 37, 101, 110, 113, and SB) within 0, 7, and 14 days (two-way ANOVA: strain: F(5,45) = 19,232, *p* = 0.0000, se ± 0.365406–0.516763; time: F(2,45) = 4.2397, *p* = 0.02056; se ± 0.349850; strain × time: F(10,45) = 5.7225; *p* = 0.00002, se ± 0.632903–0.895059). Statistical significance of the analyses is included in the Supplementary Spreadsheet S1.

ASBC Color was assessed at days 0, 7, and 14. The higher the ASBC Beer Color value, the darker the color of the beer. Light beers usually are in the range of 1.5–3.0 ASBC color units, pale and amber beers in the range of 3.0–15.0, and dark beers in the range of 15.0–200 and more [42]. The results of the color assay are presented in Figure 10.

#### 3.2.7. High-Performance Liquid Chromatography Analysis

**Figure 11:**
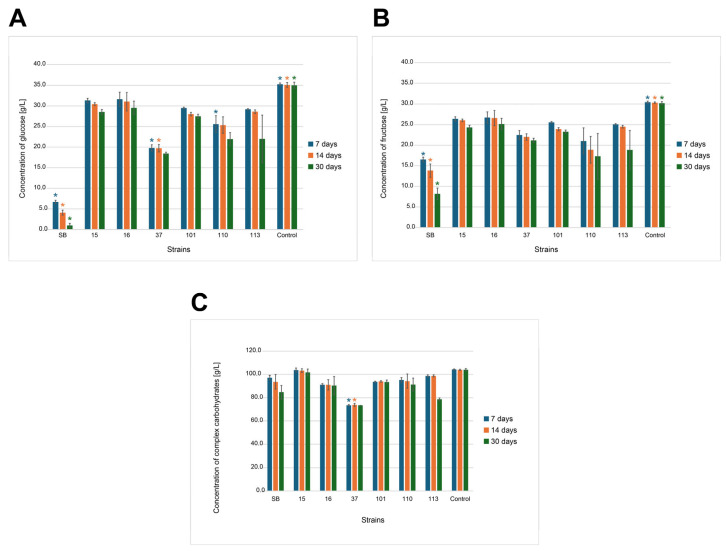
Changes in the concentration of glucose (**A**), fructose (**B**), and complex carbohydrates (**C**) (g/L) in underbeer fermented with various yeast strains (SB, 15, 16, 37, 101, 110, and 113) and a control sample after 7, 14, and 30 days of fermentation. Bars represent mean values ± standard deviation (*n* = 3). Colored asterisks (*) indicate statistically significant differences (*p* < 0.05) between all the strains and the control within the same time point (7, 14, or 30 days), as determined by Tukey’s HSD test (Supplementary Spreadsheet S2).

**Figure 12:**
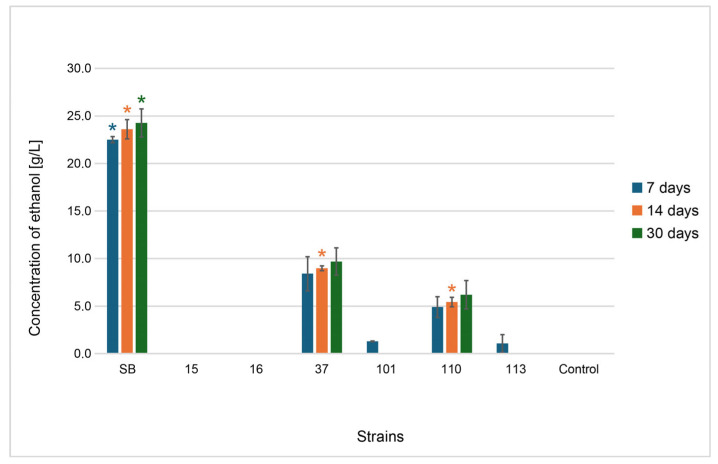
Changes in the concentration of ethanol (g/L) in underbeer fermented with various yeast strains (SB, 15, 16, 37, 101, 110, and 113) and a control sample after 7, 14, and 30 days of fermentation. Bars represent mean values ± standard deviation (*n* = 3). Colored asterisks (*) indicate statistically significant differences (*p* < 0.05) between all the strains and the control within the same time point (7, 14, or 30 days), as determined by Tukey’s HSD test (Supplementary Spreadsheet S3).

**Figure 13:**
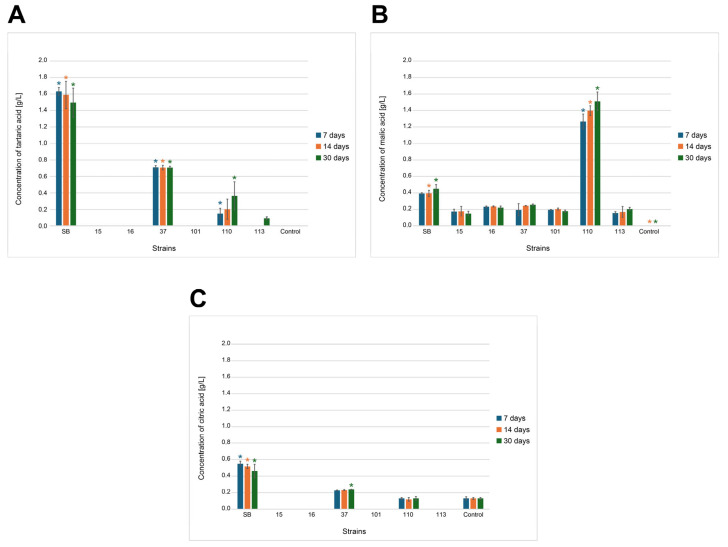
Changes in the concentration of tartaric acid (**A**), malic acid (**B**) and citric acid (**C**) (g/L) in underbeer fermented with various yeast strains (SB, 15, 16, 37, 101, 110, and 113) and a control sample after 7, 14, and 30 days of fermentation. Bars represent mean values ± standard deviation (*n* = 3). Colored asterisks (*) indicate statistically significant differences (*p* < 0.05) between all the strains and the control within the same time point (7, 14, or 30 days), as determined by Tukey’s HSD test (Supplementary Spreadsheet S3).

The wort prepared after sterilization and before inoculation contained carbohydrates: glucose (35.26 g/L) and fructose (30.42 g/L), originating from the hydrolysis of sucrose and complexed carbohydrates (104.30 g/L) containing products of partial starch hydrolysis released during grain roasting (e.g., maltodextrin) and inulin present in chicory (retention times were the same under separation conditions). The small amount of citric acid (0.13 g/L), which was a component of the mixture as an acidity regulator, was also detected. During incubation, changes in the sugar content occurred in the wort due to their assimilation by yeast cells and metabolism (including ethanol fermentation). Detailed data on substrate consumption by six strains of potentially probiotic yeasts and Sacch_boulardi are presented in Figure 11A–C. Significant changes in the composition of the wort were observed within the first days of fermentation, which depended on the yeast used. It is clearly visible that the monosaccharides glucose and fructose were used the fastest by Sacch_boulardi, at 28.68 g/L and 13.87 g/L, respectively. The strain 37_Sacch_cerevisiae assimilated 15.47 g/L glucose and 7.97 g/L fructose and was the best in assimilation of the complexed sugars. *M. pulcherrima* and *P. kudriavzevii* yeasts assimilated 16.36–27.45% of glucose and 15.94–30.86 of fructose at that time, and *H. uvarum* strains assimilated sugars the least, about 10–11% of glucose and 12–13% of fructose. The consumption of complexed carbohydrates was also low. Rapid sugar consumption was compatible with ethanol production (Figure 12), and the highest amount was produced by Sacch_boulardi, with as much as 22.51 g/L after 7 d, while 37_Sacch_cerevisiae produced 8.4 g/L of ethanol during this time and 110_Metsch_pulcherrima, 101_Pich_kudriavzevii, and 113_Metsch_pulcherrima produced 4.91 g/L, 1.29 g/L, and 1.07 g/L, respectively. Both strains of *H. uvarum* were not able to produce ethanol in the conditions used. In *Saccharomyces* yeast, an increase in citric acid concentration was observed above the concentration in the broth by 0.1 g/L and 0.42 g/L for strains 37_Sacch_cerevisiae and Sacch_boulardi, respectively (Figure 13C). Tartaric acid was also detected in small amounts during fermentation carried out by *Saccharomyces* spp., with 1.63 g/L and 0.71 g/L for Sacch_boulardi and 37_Sacch_cerevisiae, respectively, and by 110_Metsch_pulcherrima, with 0.15 g/L (Figure 13A). Malic acid appeared in the cultures of all strains in concentrations of 0.16–0.39 g/L, with the exception of 110_Metsch_pulcherrima, where 1.27 g/L was detected after 7 d of fermentation (Figure 13B). In the following weeks, when the batches were stored at refrigeration temperature, *Saccharomyces* yeast consumed glucose and fructose slowly and also produced ethanol to final concentrations of 9.69 g/L for 37_Sacch_cerevisiae and 24.27 g/L for Sacch_boulardi. Ethanol was also present throughout the duration of the experiment in the 110_Metsch_pulcherrima culture (up to 6.20 g/L). The remaining yeasts also assimilated sugars slowly but showed no metabolic activity. During the storage of samples in refrigerated conditions, stabilization of the composition of the beverages was visible (Figure 12 and Figure 13A–C).

## 4. Discussion

In the experiment, yeasts with probiotic potential were used as starter monocultures for the production of a traditional Polish fermented beverage called underbeer, which belongs to the group of low-alcohol or non-alcoholic products. The yeast strains were initially tested for tolerance to ethanol as a stress agent and their ability to produce H_2_S, as these are characteristics describing starter cultures. The analysis showed differences between strains in ethanol tolerance according to expectations for both Saccharomyces and non-Saccharomyces yeast. Taking into account *S. cerevisiae* strains, such performance is comparable to classical wine Saccharomyces strains, which commonly ferment to 12–15% ethanol concentration [43,44,45]. Results obtained for the strain Sacch_boulardi (*S. cerevisiae* var. *boulardii* CNCM I-745) align with the ethanol tolerance assay presented by Ramirez-Cota et al., 2020 [30]. In the case of non-Saccharomyces yeasts, strains 15 and 16, belonging to *H. uvarum*, showed slight growth even at 10% ethanol, which is consistent with Matraxia et al.’s, 2021, observation [17]. These strains showed very limited ethanol tolerance, in line with their known role as early-phase fermenters in wine and cider [46,47], which may depend on the incubation temperature [22]. Both *H. uvarum* strains failed to propagate vigorously once ethanol exceeded 6–8%, which matches numerous literature reports for this genus [46,47,48,49,50,51]. The strain 101_Pich_kudriavzevii showed a moderate ethanol tolerance, better than *Metschnikowia* strains but still lower than *Saccharomyces*. So, for practical purposes, this *Pichia* strain could actively grow up to around 6–8% ethanol. This behavior aligns with literature: *Pichia kudriavzevii* is often noted to tolerate around 7% ethanol in traditional fermentation environments [52]. The strain’s limit (8% with slight growth) is slightly above that typical range but still far below *S. cerevisiae*’s typical ethanol tolerance threshold [23,44]. Both of the *M. pulcherrima* strains (110 and 113) proved to be quite sensitive to ethanol, which aligns with literature observations, where *Metschnikowia* strains usually show survivability in 3–5% ethanol and are used mainly in cofermentation with *Saccharomyces cerevisiae* [53,54,55].

Another important feature of the yeast used in fermentation processes is H_2_S. This sulfur compound is of great importance in industries producing alcoholic beverages such as wine, beer, or cider. Most often, yeast produces it as a result of the reduction of inorganic sulfur compounds, most commonly from sulfides released during the decomposition of organic compounds. For example, the amount of H_2_S during beer fermentation is related to the boiling time of the wort. H_2_S was suggested to play metabolic and protective roles in the cell during the early phase of fermentative transition, where a time-critical nutritional switch and oxidative stress response occur [56]. The process of releasing this compound has been fairly well understood for Saccharomyces yeasts [57]. Our isolates 37_Sacch_cerevisiae and Sacch_boulardii also showed the ability to produce H_2_S on BIGGY agar. Positive results were obtained for all strains with the exception of *Hanseniaspora uvarum* strains and were typical for individual species [17,56,57].

The monocultures of studied yeasts were used for fermentation of the prepared wort based on roasted barley grain, chicory root and hops. Yeast viability during fermentation plays a crucial role in both fermentation efficiency and the stability of the final product, and in the context of probiotic food products, survivability of probiotic strains is a key [30,58,59,60,61]. Slight fluctuations in yeast counts observed during fermentation may be related to stress responses initiated by challenging environmental conditions during fermentation. Such stressors, like rising ethanol levels, nutrient shortages, changes in osmotic pressure, etc., trigger survival mechanisms. These include metabolism shifts and changes in gene expression levels, which can temporarily reduce yeast growth or viability. Research indicates that these stress responses are key to how yeast adapts, and they often lead to short-term declines in cell counts during fermentation [61,62,63,64,65,66]. All of the tested strains exceeded the minimum viable cell count of 10^6^ recommended by World Health Organization (WHO), which suggests their potential use in probiotic underbeer preparation in the context of survivability during fermentation [7,67]. Additionally, taking into account the size of the typical served portion of underbeer (250–500 mL), the viable cell count of 10^9^ suggested by other authors should be reached [68]. The yeast remained viable throughout the entire storage period (1 month). Our result are in agreement with Hinojosa-Avila, 2025 [13], who, during the production of beer based on mixed wort, found that commercial *S. cerevisiae* var. *boulardii* yeast reached 6.2 Log CFU/mL at the end of fermentation, whereas Mulero-Cerezo, when trying to use *S.cerevisiae* var. *boulardii* to produce probiotic craft beer, obtained a cell count of 8.3 × 10^6^ CFU/mL.

The fermentation process lasted 14 days at room temperature, after which the products were stored in a refrigerator. Fermentation conditions were standardized for all strains due to the preliminary nature of the research and the possibility of comparing yeast performance. Certainly, in order to achieve optimal results, fermentation requires optimization with respect to temperature, pH, culture time, and oxygen access, depending on the preferences of individual strains. Analyses of underbeers conducted in terms of substrate consumption, basic metabolites production, and fermentation vigor indicate the fermentative activity of the yeast. The analyses showed that Sacch_boulardii was the most active, utilizing sugars the fastest, exhibiting the highest fermentation vigor, and producing the highest concentrations of ethanol, tartaric acid, and citric acid. The next strain was the wine-derived isolate 37_Sacch_cerevisiae, which had lower activity in the wort environment but maintained a similar trend. These Saccharomyces spp. strains exhibited one of the highest levels of CO_2_ production from all tested yeasts, underscoring their vigorous fermentative capability. Such characteristics make them suitable for fermentations aimed at rapid conversion of sugars to alcohol and CO_2_, which aligns well with previous studies in the field of beer fermentations [69,70,71]. It should be emphasized that the commercial probiotic yeast *S. cerevisiae* var. *boulardii* was used by other researchers to produce probiotic beer with good results [10,12]. Silva stated that wheat and sour beers can be highlighted as useful matrices to deliver probiotic strains, and Gutiérrez-Nava confirmed the usefulness of barley wort as a potential medium to produce probiotic beverages with the yeast S. boulardii. At the opposite end of the spectrum were the *H. uvarum* strains, also derived from wine, which, however, did not perform well in the wort environment, as they consumed little sugar, had very low fermentation vigor, and did not produce ethanol. In general, *Hanseniaspora* yeasts are glucophilic in nature, but they do not completely consume the sugars in grape must. Ethanol production, on the other hand, is species-dependent, and these yeasts are mainly known for the production of aromatic compounds and acids. On the other hand, *Hanseniaspora* does not have the ability to assimilate maltose, and only a few species of this genus can utilize sucrose. Therefore, they are not considered components of the natural microbiota of grain-based fermentation, but their use is in line with the trend towards the production of low-alcohol or non-alcoholic beers [17,25]. The isolates 101_Pich_kudriavzevii, 110_Metsch_pulcherrima, and 113_Metsch_pulcherrima had slightly higher fermentation activity, especially strain 110, which produced ethanol and a lot of malic acid. Additionally, one strain of *Metschnikowia pulcherrima* (113_Metsch_pulcherrima) presented high FV at 14 d, with 6.76 g compared to only 0.44 g at day 7, which might be caused by the evolutionary adaptation of the strain during the fermentation process [62,72]. The adaptive mechanisms of *Metschnikowia* yeast to fermentation environments have been studied to some extent at various levels: genetic, cellular, and extracellular (cellular response). In-depth research by Sipiczki et al., 2024, comprehensively explains the unique adaptive abilities of these yeasts at the genetic level due to the presence of a chimeric genome structure (heterozygosity, multiple gene alleles) and gene regulation, which allows for the switching of allele expression to suit conditions and further adaptation of metabolism to the environment [73]. It is very interesting that these yeasts assimilated sugars poorly and used them mainly for growth, as evidenced by the number of live yeast cells analyzed during the experiment.

During and after fermentation, the selected underbeers’ properties were examined on the basis of beer characteristics such as pH, titratable acidity, turbidity, and color [13]. The acidity analysis was consistent with the pH and metabolite analysis, as the highest acidity and lowest pH were correlated with the highest concentrations of acids produced by the tested yeasts.

Turbidity is considered a decrease in a liquid’s transparency due the presence of undissolved substances, and turbidity is very often indicative of biomass formation, yeast suspension, and the colloidal stability of a beverage. With progressing fermentation, flocculating strains begin to sediment, reducing turbidity, while non-flocculating strains may maintain high levels of suspended cells. However, increased turbidity over time is not always solely attributable to biomass growth. Other contributing factors include the accumulation of extracellular polysaccharides, mannoproteins, and β-glucans released during autolysis or cell wall remodeling [74,75]. These macromolecules can increase colloidal instability and haze formation in the liquid phase. Additionally, mannoproteins might provide various prohealth properties such as prebiotic, antimicrobial, immunostimulating, and antioxidant activities [76]. The release of vesicles and exometabolites into the medium during late fermentation stages can affect turbidity levels [74,77,78]. The level of turbidity may vary during incubation, and decreases in its value may indicate the ability of a yeast to form aggregates and sediments at the bottom of fermentation vessels, as was observed for 101_Pich_kudriavzevii and Sacch_boulardi at 14 days. Such an observation could result from increased flocculation activity or partial cell sedimentation during the final fermentation stage, which might be connected with reduced metabolic activity of yeast and lower amount of excreted extracellular metabolites, which can also affect the turbidity [69]. Additionally, autolysis processes might lead to partial clarification as larger colloidal particles settle or degrade [79].

All yeast strains increased the titratable acidity (TTA) of the beverage during fermentation, which is a typical outcome due to the production of organic acids by yeast metabolism [80,81]. Both *Saccharomyces* spp. strains (37_Sacch_cerevisiae and Sacch_boulardi) exhibited the highest TTA, reflecting their robust metabolic activity and known acidification capacity. This high level of acid production is consistent with their role in wine and beer fermentation, where they generate various acids such as acetic, succinic, and lactic acids as byproducts of their metabolism [81,82,83]. Strains 15_Hans_uvarum and 16_Hans_uvarum showed low increases in acidity during the whole fermentation period, which is consistent with the observations of other authors where *H. uvarum* contributes to acidification through limited acetic acid production that could enhance freshness perception in fermented beverages [50,84,85]. Both *M. pulcherrima* strains (110_Metsch_pulcherrima and 113_Metsch_pulcherrima) presented values of TTA between those of the tested strains belonging to *Hanseniaspora* and Saccharomyces. This observation is consistent with prior studies showing that *M. pulcherrima* produces relatively fewer organic acids, partly due to its lower fermentative capacity and slower metabolism [53,72,86,87,88].

The color of all tested samples changed throughout the experiment and did not show any trend or tendency related to the species or fermentation period, which allows us to assume that this is a strain-specific feature of the tested yeasts. All underbeers were classified as pale and amber beers, taking into account EBC values. However, the following trends can be observed by analyzing the obtained results: (1) Gradual color reduction (strain 15_Hans_uvarum): characterized by a consistent decline in EBC values across time points. This may reflect steady polyphenol adsorption or enzymatic transformation processes, often associated with yeasts’ metabolic activity [81,89]. (2) Gradual color increase (strain 16_Hans_uvarum): characterized by a consistent increase in EBC values across time points, which may result from progressive pigment release from ingredients due to mild enzymatic activity or structural disruption of material matrices. Moreover, limited pigment adsorption to yeast cell walls and continued extraction in a low-ethanol environment could explain this rise [89,90,91]. (3) Initial increase followed by decline (strains 101_Pich_kudriavzevii and 110_Metsch_pulcherrima): those two strains showed a temporary rise in color intensity before a drop. This could be due to delayed autolysis, cell wall disruption, or pigment release followed by precipitation or adsorption of color-related substances by [92,93]. (4) Initial decrease followed by increase (strains 37_Sacch_cerevisiae, 113_Metsch_pulcherrima, and Sacch_boulardi): such a pattern may reflect early adsorption of polyphenols onto yeast cell walls or enzymatic transformation, reducing color initially. In later stages, autolysis or changes in cell surface chemistry may result in the release of previously bound pigments into the medium [92,93].

Our study is part of the current trend of searching for optimal food matrices for probiotic and potentially probiotic yeasts in order to obtain functional products. In recent years, there has been an intense increase in research on the use of unconventional yeasts, e.g., *Pichia*, Debaryomyces, Kluyveromyces, *Metschnikowia*, and others, for the production of fermented beverages such as low-alcohol and non-alcoholic beers, ciders, kombucha, and other plant-based fermented beverages [1,2,6,8]. The results presented here are only a preliminary step towards further research, which can be conducted in many ways: (1) the use of yeast monocultures or yeast co-cultures or yeast-bacterial co-cultures, (2) the addition of health-promoting additives, e.g., prebiotics; (3) the optimization of fermentation conditions and upscaling. These research proposals have a chance of being implemented, as market trends in recent years are optimistic. According to Research and Market, the global probiotics market was worth approximately USD 59.3 billion in 2022, and estimates predict strong growth to approximately USD 91.7 billion by 2030. Bacteria are the main segment of probiotics, but the yeast segment is expected to see dynamic growth over the next 8 years [2]. According to an analysis by Future Market Insight, the probiotic beverage market will grow from USD 22,069 million to USD 43,009 million by 2035, reflecting consumers’ ever-increasing awareness of health and well-being and the prevention of many diseases [94]. The functional beverage category is expanding through the introduction of, among other things, dairy-free options, i.e., plant-based alternatives. The growing interest of societies on all continents in functional probiotic beverages is forcing the adaptation of appropriate global legal regulations for manufacturers, as can be learned from a detailed article by Mukherjee et al., 2022 [95]. In summary, in the context of the research conducted, it is certainly worthwhile to continue it by following the latest market trends and consumer expectations.

## 5. Conclusions

All of the tested probiotic yeast strains presented the ability to conduce underbeer fermentation. None of the tested strains presented properties above expectations for already-examined strains belonging to the same genus in the context of beverage fermentation. However, due our previous findings regarding the probiotic potential of the yeast strains used in the study and the inulin presented in underbeer wort, the study can be a starting point for further research on aspects of the production of both probiotic and synbiotic beverage preparations, as well as an impulse to search for often-forgotten regional products that can be an interesting base for probiotic products.

## Figures and Tables

**Table 1:** Strain details.

Strain Number	Strain Species	Strain Code ^1^
15	*Hanseniaspora uvarum*	15_Hans_uvarum
16	*Hanseniaspora uvarum*	16_Hans_uvarum
37	*Saccharomyces cerevisiae*	37_Sacch_cerevisiae
101	*Pichia kudriavzevii*	101_Pich_kudriavzevii
110	*Metschnikowia pulcherrima*	110_Metsch_pulcherrima
113	*Metschnikowia pulcherrima*	113_Metsch_pulcherrima
SB	*Saccharomyces cerevisiae* var. *boulardii*	Sacch_boulardi

^1^ Strain codes used in our previous articles [9,10] where the probiotic potential of the strains was tested.

## Data Availability

Data will be made available on request.

## Supplementary Materials

The following supporting information can be downloaded at: https://www.mdpi.com/article/10.3390/foods14162921/s1, the Supplementary Spreadsheet S1 (Tukey tests for Figure 1, Figure 2, Figure 3, Figure 4, Figure 5, Figure 6, Figure 7, Figure 8, Figure 9 and Figure 10): Supplementary_1.xlsx; Supplementary Spreadsheet S2 (Tukey test for Figure 11): Supplementary_2.xlsx; Supplementary Spreadsheet S3 (Tukey tests for Figure 12 and Figure 13): Supplementary_3.xlsx.
